# Age-related outcomes in MSI/dMMR gastrointestinal cancers treated by immune checkpoint inhibitors and toxicity’s impact on efficacy: an immunoMSI cohort study

**DOI:** 10.1016/j.esmogo.2024.100047

**Published:** 2024-04-30

**Authors:** L. Mailly-Giacchetti, R. Colle, T. Samaille, D. Lopez-Trabada Ataz, L. Faucheux, A. Duval, T. Andre, R. Cohen

**Affiliations:** 1 Department of Medical Oncology, Saint-Antoine Hospital, AP-HP, Sorbonne Université, Paris, France; 2 Sorbonne Université, Paris, France; 3 Sorbonne Université, Unité Mixte de Recherche Scientifique 938 et SIRIC CURAMUS, Centre de recherche Saint-Antoine, équipe instabilité des microsatellites et cancer, équipe labellisée par la Ligue Nationale contre le cancer, Inserm, Paris, France

**Keywords:** immune checkpoint inhibitors, adverse events, microsatellite instability, metastatic gastro-intestinal tumor, elderly

## Abstract

**Background:**

Immune-checkpoint inhibitors (ICIs) are the standard of care for microsatellite instability (MSI) metastatic gastrointestinal cancer (mGIC) patients in first- and later-treatment lines. We compared tolerability and efficacy of ICIs in elderly (aged ≥75 years) versus non-elderly MSI mGIC patients and analyzed the correlation between immune-related adverse events (irAEs) and efficacy.

**Patients and methods:**

This single-center prospective cohort study included MSI mGIC patients treated with ICIs, excluding chemotherapy. Assessments covered grade ≥3 irAEs and ≥2 endocrine irAEs (E-irAEs).

**Results:**

Among 201 patients, 24 were elderly (mean age 75–90 years) and 177 non-elderly (mean age 22-74 years). In the overall population, grade ≥3 irAEs and E-irAEs incidence was 40% with the anti-programmed cell death protein 1 + anti-cytotoxic T lymphocyte-associated antigen 4 and 23% with anti-programmed cell death protein 1 monotherapy (*P* = 0.011). Treatment combination was administered to 29% of elderly and 40% of non-elderly patients. The incidence of grade ≥3 irAEs and E-irAEs was 37%/29% with monotherapy (*P* = 0.48) and 57%/39% with combination (*P* = 0.43) in elderly/non-elderly patients. No significant difference was observed in progression-free survival [hazard ratio (HR) = 1.15, 95% confidence interval (CI) 0.57-2.32, *P* = 0.7] and OS (HR = 1.61, 95% CI 0.75-3.43, *P* = 0.25) between elderly and non-elderly. Cox regression analysis with a time-dependent variable showed no survival difference between patients with/without grade ≥3 irAEs and E-irAEs (progression-free survival: HR = 1.19, 95% CI 0.64-2.19, *P* = 0.59; overall survival: HR = 0.91, 95% CI 0.44-1.92, *P* = 0.81). A positive association was found, however, between objective response rate and immune treatment-related adverse event occurrence [77%/59%, immune treatment-related adverse event patients/others (*P* = 0.0012)].

**Conclusion:**

This study reveals comparable tolerability and efficacy of ICIs in elderly and non-elderly patients with MSI mGIC. Survival outcomes did not differ significantly between patients with and without grade ≥3 irAEs and E-irAEs.

## Highlights

• ICIs tolerability and efficacy are comparable in elderly and non-elderly with MSI/dMMR GIC.
• There were no survival differences observed between irTRAE and ICIs efficacy.
• ICIs efficacy and tolerability should be better evaluated in elderly patients in the future with oncogeriatric assessments.

## Introduction

A deficiency in the mismatch repair (MMR) system underlines the development of microsatellite instability (MSI) and the occurrence of a high tumor mutational burden. Notably, a pronounced peritumoral lymphocyte infiltration in MSI cancers has been documented in the literature.^1^ These characteristics are predictive of response to immune checkpoint inhibitors (ICIs). In colorectal cancer (CRC), 15% cases with localized CRC, 5% of metastatic stage (mCRC), 8%-20% of gastric cancers have an MSI phenotype.^2^, ^3^, ^4^ Most of these cases are sporadic and primarily caused by hypermethylation of MLH1 (80%). Notably, sporadic MSI phenotype tends to increase with age, particularly after 70 years. Two randomized studies, the KEYNOTE-177^5^ and Samco,^6^ demonstrated the efficacy of ICIs compared with chemotherapy with/without targeted therapy in MSI mCRC, using pembrolizumab in the first line and avelumab in the second line.^5^^,^^6^ Noteworthy overall response rates were observed in patients with advanced disease refractory to chemotherapy with/without targeted therapy, as evidenced by studies such as CheckMate-142 and KEYNOTE-164 for MSI mCRC.^7^^,^^8^ Efficacy of ICIs was then demonstrated for other MSI cancers. In their *post hoc* analysis of three trials (KEYNOTE-059, 061 and 062) Chao et al.^9^ showed superiority of pembrolizumab monotherapy or combined with chemotherapy for MSI-H gastric and gastroesophageal junction (GEJ) cancers in first-, second- or third-line treatment. In the CheckMate 649 comparing nivolumab-chemotherapy versus chemotherapy for advanced gastric, GEJ cancers and esophageal adenocarcinoma in first line, patients with MSI-H tumors treated by a nivolumab-based regimen had better overall survival (OS) regardless of programmed death-ligand 1 (PD-L1) status with a hazard ratio (HR) of 0.37 (0.16-0.37).^10^ Finally, in a large cohort of patients with MSI-H non-colorectal cancer (21% endometrial, 10.4% gastric) Marabelle et al.^11^ showed efficacity of pembrolizumab on the whole cohort with an objective response rate (ORR) of 34.8%. Based on these results, the Food and Drug Administration (FDA) and the European Medical Agency (EMA) have approved pembrolizumab as a first-line treatment in patients with MSI mCRC and the combination of nivolumab and ipilimumab for those with refractory disease to standard chemotherapy. Pembrolizumab has also received approval from the EMA for MSI unresectable or metastatic gastric, small intestine or biliary cancer in patients who experienced disease progression on treatment or following at least one prior therapy.

**Figure 1:**
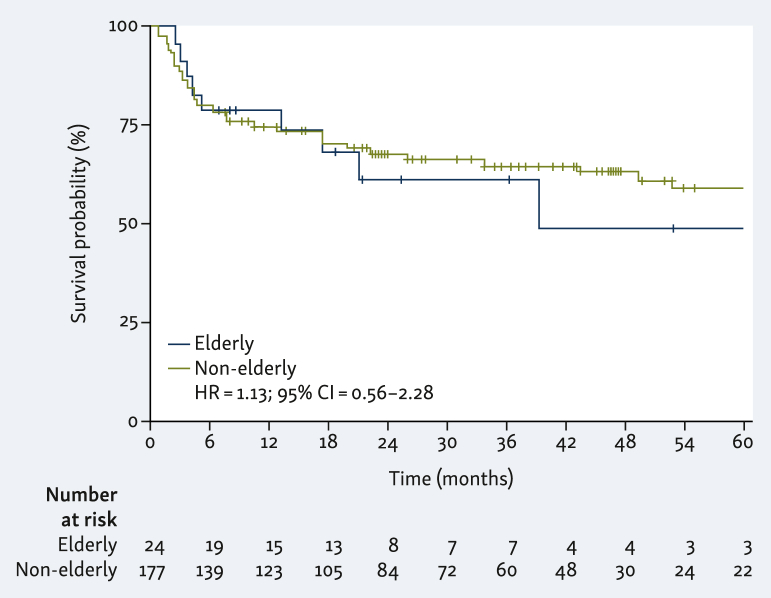
**Progression-free survival according to age.** CI, confidence interval; HR, hazard ratio.

Despite the fact that ICIs are standard of care in MSI mCRC and the increasing prevalence of this phenotype with age, there are limited data about the safety and efficacy of ICIs in elderly patients. The aging-associated immune changes render older patients more vulnerable to infections, less responsive to vaccines, and with a higher prevalence of cancers. Recent research by Goronzy and Weyand described two major immune phenomena, inflammaging and immunosenescence, in elderly individuals.^12^ Inflammaging is a pro-inflammatory state, leading to a senescent-associated secretory phenotype of monocytes.^13^ Immunosenescence has an impact on both the adaptive and innate immune systems. It is characterized by a reduction in the number of naive T cells, coupled with a reduction in their T-cell receptor diversity due to shortened telomeres. Conversely, there is an increase in memory and regulatory T cells. The diminished production of B precursors leads to a decrease in naive B lymphocytes, whereas memory B lymphocytes show a numerical increase. This shift contributes to an increased presence of auto-antibodies and a decrease in response to new antigen, as illustrated in Figure 1.^12^ These phenomena align with 12 hallmarks of aging recently described by López-Otín et al.^14^ Ferrara et al.,^15^ recently validated a senescent immune phenotype (SIP, marked by >39.5% of CD28− CD57+ KLRG1+ among circulating CD8+ T cells), which correlates with a lower response to ICI. Notably, SIP is not associated with age.^15^ These considerations raise questions about the tolerability and efficacy of ICIs in elderly patients, especially given the underrepresentation of these patients in clinical trials. From 2004 to 2015, elderly patients represented 4% to 8% of the population in the studies, with <10% of the participants being aged ≥70 years, and the median age often falling below 64 years.^16^, ^17^, ^18^ Moreover, there is a lack of oncogeriatric score specific to ICIs in comparison to chemotherapy. Tolerability and efficacy of ICIs in elderly patients emerge as a public health concern. Retrospective studies evaluating the safety and efficacy of ICIs in older patients have been published,^16^^,^^19^, ^20^, ^21^, ^22^, ^23^, ^24^, ^25^ and whereas the results are generally reassuring, in the majority of these studies, age was not associated with higher rates of immune-related adverse events (irAEs). These studies, however, neither included metastatic MSI/dMMR cases and nor the combinations of ICI(s).

The debate around immune treatment-related adverse events (irTRAEs) and their correlation to response and survival remains inconclusive. A correlation between irTRAEs and a strong immune response was shown, making it a predictive factor of better response to ICIs.^26^ While some studies, however, reported a positive association between irTRAEs and survival,^27^, ^28^, ^29^ others presented contradictory results.^30^, ^31^, ^32^, ^33^ The challenge includes the immortal-time bias, requiring adequate statistical methodology and a consequent number of events and subjects to maintain power of the analysis. Recently, Kfoury et al.^34^ published the first observational real-world cohort study on the association between irTRAEs and survival. The study used a dual analysis approach, using both a landmark analysis at 12 weeks and Cox regression with a time-dependent variable. Their findings revealed no association in the landmark analysis, but the occurrence of irTRAEs (grade ≥2) showed a significant association with survival [HR 0.56, 95% confidence interval (CI) 0.41-0.75, *P* = 0.0001] and progression-free survival (PFS, HR 0.63, 95% CI 0.47-0.83, *P* = 0.001) in a Cox regression model.^34^ These studies, however, also did not establish a conclusion regarding the association of irTRAE and survival. It is important to underline that none of these studies included MSI/dMMR cancers or the combination of ICIs.

Here, we report the first real-world evaluation of the safety and efficacy of anti-PD(L)1 ± anti-cytotoxic T lymphocyte-associated antigen 4 (CTLA4) in elderly patients aged ≥75 years with MSI metastatic gastrointestinal cancers (mGIC). Additionally, we explore the association between irTRAEs and efficiency in the whole cohort.

## Patients and methods

### Study cohort

This is a single-center, prospective immunoMSI cohort study. Patients were treated by anti-PD(L)1 and/or anti-CTLA4 without concomitant chemotherapy for mGIC at Saint-Antoine Hospital (Paris, France) between September 2014 and November 2021. All consecutive patients aged >18 years with MSI/dMMR mGIC treated with anti-programmed cell death protein 1 (PD1) monotherapy or anti-PD1 plus anti-CTLA4 combination, at Saint-Antoine Hospital, were included. Patients received treatment in the first, second, or subsequent lines of therapy. The diagnosis of MMR status was established by immunohistochemistry for deficient MMR (dMMR) and/or polymerase chain reaction (PCR) for MSI, either at the referent patients’ hospital or at Saint Antoine. This study has been approved by the local ethic committee (N_2020 - CER 2020-6).

### Outcomes

The objective of the study was to assess the safety, tolerability and efficacy of ICIs in patients aged ≥75 years (elderly) compared with those who are younger (non-elderly). The primary outcome measure was the rate of irTRAE grade ≥3 or grade ≥2 endocrine irTRAE (E-irTRAEs). Secondary endpoints were PFS, overall survival (OS) between the two age-based groups and the association between irTRAE and the efficacy (PFS, OS and ORR) of ICIs in the whole cohort.

### Adverse events and survival

All adverse events occurring during treatment were recorded and graduated according to the Common Terminology Criteria for Adverse Events (CTCAE) version 5.0. The safety assessment focused on irTRAEs grade ≥3 or E-irTRAEs grade 2. *De novo* dysthyroid disorders were considered immune-related. For colitis and dermatologic adverse events, irTRAEs were deemed related to treatment based on histological proof whereas for other events, the determination was made in consultation with immunologists, neurologists, hepatologists, or endocrine specialists. The management of irTRAEs including administration of corticosteroids, immunosuppressive agents, or discontinuation of treatment, was recorded. Time to onset of an irTRAE was defined as the time between the first dose of treatment and the onset of the earliest irTRAE. Time to resolution was defined as the time from onset to complete resolution or improvement to baseline. Permanent irTRAEs (requiring lifelong endocrine substitution therapies) were excluded from the time to resolution. Efficacy assessments included PFS, OS and ORR. PFS was defined as the time from the first dose to the first documented progression or death from any cause, whichever occurred first. OS was defined as time from the first dose to death from any cause. ORR was defined as the number of patients with a complete response (CR) plus partial response (PR) per Response Evaluation Criteria in Solid Tumors (RECIST) version 1.1 divided by the number of treated patients.

### Statistical analysis

Analysis of patient characteristics and safety data was descriptive. Quantitative variables were described as mean, median, standard-deviation and interquartile range, whereas qualitative variables were described as percentage and effective. Association between two quantitative variables was determined by the Fisher exact test, and comparison between two groups was assessed by the Wilcoxon test. Safety analysis was carried out with a logistic regression model. Associations between age and irTRAE and age and survival were analyzed through univariate analysis and then adjusted Cox multivariate analysis on parameters with *P* value <0.2 in univariate. Survival analysis was carried out using a Cox regression model, and the results presented with Kaplan–Meier curves. Log-linearity and risk proportionality were verified. To address immortal bias, the occurrence of an irTRAE was considered as a time-varying variable and analyzed with a Cox regression model with time-variable dependence. Association between irTRAE and survival was determined with a Cox regression time varying variable. Correlation between irTRAE and ORR was evaluated using the Fisher exact test and between the type of treatment (monotherapy or combination), irTRAE, and ORR was assessed by logistic multivariate regression. All analyses were carried out using R Studio software.

## Results

### Population

From 30 September 2014 to 30 November 2021, a total of 201 patients were included in the study. The median follow-up was 24 months, and at the data cut-off, 67% of patients were still alive. Patient characteristics are presented in Table 1. Among them, 24 (12%) were aged ≥75 years and 177 (88%) were aged <75 years. The median age for the whole cohort was 59 years, ranging from 22 to 74 years. In the two age groups, the median ages were 78 years (range 75-81 years) for the elderly group and 57 years (range 56-64 years) for the non-elderly group. The elderly group had a higher proportion of women, accounting for 75% compared with 42% in the non-elderly group (*P* = 0.004). The primary tumors were predominantly mCRC (*n* = 171) with less frequent occurrence of gastric or esogastric junction cancers (*n* = 13). Other primary tumors (*n* = 17) included small intestine, biliary tract and pancreas. One patient had ovarian MSI cancer. In 201 cases, 39 (19%) exhibited a hypermethylation of *MLH1* promotor. Of those, seven were aged ≥75 years. A total of 5 out of 12 (43%) gastric tumors had hypermethylated *MLH1* promotor, compared with 20% (34 out of 171) colorectal tumors. In terms of treatment, 122 patients (61%) received monotherapy, whereas 99 patients (39%) received combination therapy. Elderly patients were less likely to receive treatment in third line than their younger counterparts (20% versus 46%, respectively).

**Table 1:** Patient characteristics.

	All cohort (*N* = 201)	Elderly (*n* = 24)	Non-elderly (*n* = 177)
Age, median age, years (Q1-Q3), *n* (%)	59 (47-68)	78 (77-81)	57 (46-64)
Male, sex^a^, *n* (%)	108 (54)	6 (25)	103 (58)
Primary tumor, *n* (%)			
Colorectal	171 (79)	19 (79)	152 (86)
Gastric or esogastric junction	13 (6)	4 (17)	9 (5)
Other primary (small intestine, biliary tract and pancreas, one ovarian MSI cancer)	17 (8)	1 (4)	16 (9)
Line of treatment^b^, *n* (%)			
First-line	44 (22)	7 (29)	37 (21)
Second-line	70 (35)	12 (50)	58 (33)
≥Third-line	87 (43)	5 (20)	82 (46)
Type of treatment, *n* (%)			
Anti-PD(L)1 monotherapy	123 (61)	17 (71)	106 (60)
Combination of anti-PD(L)1 and anti-CTLA4	78 (39)	7 (29)	71 (40)
MSI/dMMR diagnosis method, *n* (%)			
PCR	133 (66)	16 (67)	117 (66)
IHC	200 (99)	23 (96)	177 (100)
Hypermethylation of *MLH1* promotor	39 (19)	7 (29)	32 (18)

CTLA4, cytotoxic T lymphocyte-associated antigen 4; dMMR, deficient mismatch repair; IHC, immunohistochemistry; MSI, microsatellite instability; PCR, polymerase chain reaction; PD1, programmed cell death protein 1, PDL1, programmed death-ligand 1; Q, quartile.

a *P* = 0.004, more female in the elderly group.

b Comparison of first-line + second-line and ≥third-line, *P* = 0.027 (Fisher exact test).

### Safety according to age

Sixty (30%) patients had at least one irTRAE grade ≥3 or E-irTRAE grade 2. Among them, 32 (53%) patients were treated with the combination of anti-PD1 + anti-CTLA4, whereas 28 (47%) patients received monotherapy (*P* = 0.011, Fisher exact test; Table 2). There was no significant difference between the two age groups: 9 (37%) elderly patients and 51 (29%) non-elderly patients had irTRAE (*P* = 0.48, chi-square test; Table 2). Elderly patients treated with the combination did not exhibit a higher incidence of irTRAEs compared with non-elderly patients [four (57%) versus 28 (39%), respectively, *P* = 0.43]. irTRAEs occurring in elderly patients under the combination therapy included hypothyroidism, corticotropic, and antehypophyseal insufficiency, and immune pancreatitis. The most frequent irTRAEs were endocrine-related toxicities (39%) with 12 cases of hypothyroidism and 12 cases of corticotropic insufficiencies. Only three E-irTRAEs were of grade 3 involving immune-induced diabetes, corticotropic failure and antehypophyseal insufficiency. The most common irTRAEs of grade ≥3 were increased transaminase levels (19%) and colitis (10%). One irTRAE of grade 5 was reported, depicting acute hepatocellular failure due to hepatic infarction in a patient treated for a cholangiocarcinoma. All immune adverse events related to treatment are described in Table 3. A total of 16 (27%) patients presented more than one irTRAEs, but none of these cases involved elderly patients. The median time to the onset of irTRAEs was 4 months, with a very heterogenous time frame to onset of the first irTRAE per patient (Supplementary Figure S1, available at https://doi.org/10.1016/j.esmogo.2024.100047). The median time to resolution was 8 weeks, with E-irTRAE necessitating lifelong substitution therapies, exhibiting a median duration of 4 months (a mean of 6.5 months, Q1-Q3: 2.3-8.4 months).

**Table 2:** Incidence of irTRAEs according to treatment and age.

	irTRAEs	No irTRAEs	Total
Combination anti-PD-1 + anti-CTLA4, *n* (%)	32 (40)	47 (59)	79 (39)
Monotherapy *n* (%)	28 (23)	94 (77)	122 (61)
Total∗, *n* (%)	60 (30)	141 (70)	201 (100)
Non-elderly	51 (29)	126 (71)	177 (88)
Elderly	9 (37)	15 (62)	24 (22)
Total∗∗, *n* (%)	60 (30)	142 (70)	201 (100)

CTLA4, cytotoxic T lymphocyte-associated antigen 4; irTRAE, immune treatment-related adverse event; PD-1, programmed cell death protein 1.

∗ *P* = 0.011 (Fisher exact test).

∗∗ *P* = 0.48 (chi-square test).

**Table 3:** irTRAEs characteristics.

irTRAEs	All cohort (*N* = 201)	Elderly (*n* = 24)	Non-elderly (*n* = 177)
Grade ≥3, *n* (%)	**51 (64)**	**6 (62)**	**45 (64)**
Hepatic, *n (%)*	18 (35)	2 (33)	16 (38)
Increased transaminases	14	2	12
Cholestasis	3	0	3
Hepatocellular failure	1	0	1
Digestive, *n* (%)	12 (23)	1 (17)	11 (22)
Colitis	8	0	8
Pancreatitis	4	1	3
Endocrine, *n* (%)	3 (6)	0	3 (7)
Corticotropic insufficiency	1	0	1
Diabetes	1	0	1
Hypophysitis	1	0	1
Other^a^	18 (35)	3 (50)	15 (33)
Grade 2 endocrine	**28 (36)**	**3 (37)**	**25 (36)**
Dysthyroidism	14	1	13
Corticotropic failure	11	0	10
Thyrotropic insufficiency	1	1	1
Hypophysitis	2	1	1

irTRAEs, immune treatment-related adverse events.

a (*n* = 1) bullous pemphigoid, necrotizing myositis, (*n* = 3) autoimmune thrombocytopenias, (*n* = 2) diffuse interstitial lung diseases, (*n* = 2) sarcoidosis-like conditions, anterior uveitis, aseptic meningitis, severe left ventricular dysfunction, acute polyradiculoneuritis, renal failure, (*n* = 2) septicemias, Sjogren’s-like syndrome and rheumatoid arthritis.

### Management of irTRAEs

Within the entire cohort, 33 events (42%) led to discontinuation of treatment. Five (21%) elderly patients and 23 (13%) non-elderly patients permanently discontinued treatment due to irTRAEs. There was no significant difference between the two age groups (*P* = 0.72). Overall, 38 irTRAEs necessitated immunosuppressive corticosteroid treatment. There was no difference in management of irTRAEs according to age (4 elderly patients and 34 non-elderly patients). Two non-elderly patients were treated with immunomodulator agents (i.v. immunoglobulin and anti- tumor necrosis factor) for myocarditis and colitis.

### Efficacy according to age

In the univariate analysis, type of treatment (monotherapy/combination anti-PD1 + anti-CTLA4) was the only parameter with *P* value <0.02. In multivariate analysis adjusted for the type of treatment, however, no significant association was found between OS, PFS and age (*P* = 0.24 and *P*= 0.96, respectively, Table 4).

**Table 4:** Multivariate analysis of age and survival.

	PFS			OS		
	HR	95% CI	*P* value	HR	95% CI	*P* value
Age, years						
<75	—	—		—	—	
≥75	0.98	0.49-1.99	0.96	1.61	0.73-3.52	0.24
Monotherapy						
No	—	—		—	—	
Yes	2.83	1.61-4.97	**<0.001**	3.36	1.69-6.66	**<0.001**
Line of treatment						
First-line				—	—	
Second-line				3.09	1.05-9.10	**0.040**
Third-line				3.79	1.30-11.0	**0.014**

Bold indicates statistically significant.

CI, confidence interval; HR, hazard ratio; OS, overall survival; PFS, progression-free survival.

**Figure 2:**
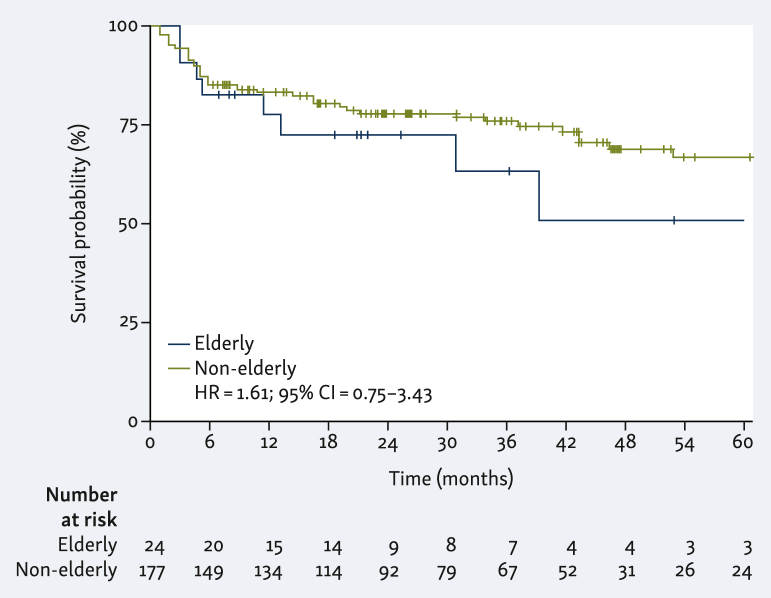
**Overall survival according to age.** CI, confidence interval; HR, hazard ratio.

Neither the median PFS nor the median OS were reached in the entire cohort nor in the non-elderly group. In the elderly group, the median PFS was 39.2 months and the median OS was not reached (PFS: HR 1.13, 95% CI 0.56-2.28, *P* = 0.7; OS: HR 1.61, 95% CI 0.75-3.43, *P* = 0.25; Figures 1 and 2, Supplementary Figure S2, available at https://doi.org/10.1016/j.esmogo.2024.100047). In the entire cohort, ORR was 64%, and CR 28%. There was no significant statistical difference according to age (*P* = 0.26, Fisher exact test; Supplementary Table S1, available at https://doi.org/10.1016/j.esmogo.2024.100047).

### Efficacy according to the occurrence of irTRAEs

For patients without irTRAEs, the median PFS was 43.2 months, and the median OS was 52.8 months. In the cohort of patients who experienced irTRAEs, neither the median PFS nor the median OS were reached. There was no statistical difference between the two groups of patients (PFS: HR 1.19, 95% CI 0.64-2.19, *P* = 0.59; OS: HR 0.91, 95% CI 0.44-1.92, *P* = 0.81).

Among 60 patients with irTRAEs, 46 (77%) achieved objective response, with 19 (32%) experiencing CR. This demonstrated a significant statistical difference (*P* = 0.001, Fisher exact test) compared with patients without irTRAEs (Supplementary Table S2, available at https://doi.org/10.1016/j.esmogo.2024.100047).

## Discussion

Today ICI(s) are the standard of care for the treatment of MSI/dMMR mGIC. There is, however, a lack of data on toxicities in the elderly, especially with combination treatments. In our study, we conducted a two-age cohort analysis of patients treated with ICI(s) monotherapy (61%) and combination therapy (39%) for an MSI/dMMR mGIC, using a cut-off of 75 years old to distinguish between elderly and non-elderly. No statistically significant difference was found in irTRAEs grade ≥3 or E-irTRAEs grade 2 between elderly and non-elderly patients (*P* = 0.48). In the entire cohort, patients treated with combination therapy had a higher incidence of irTRAEs than those receiving monotherapy (40% and 23% respectively, *P* = 0.011). Importantly, elderly patients treated with combination therapy did not experience more irTRAEs compared with non-elderly (57% and 39%, respectively, *P* = 0.43). No significant differences were observed based on the type of irTRAE in the elderly versus non-elderly population. In terms of survival outcomes, neither median PFS nor median OS was reached for the entire cohort or the non-elderly group, whereas in the elderly group, median PFS was 39 months and median OS was not reached. There was no significant difference between the elderly and non-elderly groups in terms of PFS and OS. These results are concordant with existing literature, such as these reported in the KEYNOTE-177, CheckMate-142 and KEYNOTE-059, 061 and 062 (for gastric cancers) studies, where median OS was not reached. Some 22% of patients had irTRAEs grade ≥3 in KEYNOTE-177. In the KEYNOTE-158 trial, median OS was 23.5 months and 15% of patients had irTRAEs grade ≥3.^5^^,^^11^^,^^35^ Similar results have been described in retrospective and real-life cohort studies, however, in most of these studies, the cut-off age was set at 70 years, and none of them included combination therapies or MSI/dMMR cancers.^36^

Our study had several limitations, including its retrospective and monocentric nature, along with a small sample size of elderly patients (*n* = 24). This limited number of elderly patients is one of the major biases in our study. Although the observed OS difference did not reach statistical difference, the HR was 1.61, and the appearance of the curves seemed to dissociate in favor of the non-elderly group. The low number of elderly patients in the cohort is responsible for a drop in power that must be emphasized. Thus, we cannot eliminate a false negative on OS. Another bias in our study is that all patients, particularly the older ones, exhibited a performance status of 0 or 1, as the majority of treatment with ICI(s) occurred within clinical trials, where patients with PS ≥1 were excluded, and geriatric evaluations were not conducted in our study. Consequently, our results cannot be extrapolated to a population of elderly and frail patients. The geriatric population is recognized for its heterogeneity, with performance status emerging as a better predictive marker than chronological age. The G8 score, a short screening test for frailty in elderly patients treated with cytotoxic chemotherapy, is recommended before determining a therapeutic strategy. Based on the G8 score results, patients are addressed to geriatric physicians for comprehensive geriatric assessment.^37^^,^^38^ Whereas other scores have been approved,^39^, ^40^, ^41^ they were all validated for cytotoxic chemotherapy and not for ICI(s) treatments, which have distinct mechanisms and adverse events. There is a real need to evaluate and use oncogeriatric scores for ICI(s) treatments. In our study, only irTRAEs grade ≥3 or E-irTRAEs grade 2 were analyzed to avoid recruitment bias and consider their impact on quality of life. No significant differences were observed between the two aged-based groups regarding the management of irTRAEs, including actions such as discontinuation or the administration of immunosuppressive corticosteroids. Administration of corticosteroids can lead to metabolic, cardiovascular and psychiatric complications. Therefore, particular attention should be exercised when administering them to elderly patients. Replacement with immunomodulators or other immunosuppressive agents may be an option worth discussing with geriatric and immunologist physicians.

One of the objectives of our analysis was to assess the association between irTRAEs and the efficacy of ICIs in the whole cohort. Employing a time-dependent variable Cox regression allowed us to mitigate the risk of immortal bias and revealed no disparities in survival outcomes. A positive association emerged, however, between ORR and the occurrence of irTRAEs: 77% of patients in the cohort with irTRAE (32% of CR) compared with 59% (27% of CR) in the remining group (*P* = 0.0012). This association remained significant even after adjusting for the type of treatment (monotherapy or combination) with an odds ratio of 2.64 (95% CI 1.20-6.22, *P* = 0.021). This association has been a focal point of recent research, especially in advanced non-small-cell lung cancer and melanoma. In a meta-analysis, Fan et al.^31^ showed a better response in patients with irTRAEs, reporting an odds ratio of 3.91 for ORR and enhanced survival outcomes reflected in both PFS and OS (HR 0.54 and 0.51, respectively). It is important to note, however, that the statistical analysis was limited to a landmark analysis, and all grade irTRAEs were recorded.^31^ Recently, Socinski et al.,^42^ analyzed pooled data from three clinical trials (IMpower130, IMpower132, IMpower150) evaluating the combination of chemotherapy plus atezolizumab in the first-line setting of metastatic non-small-cell lung cancer. To avoid immortal bias, the authors carried out the landmark analysis at 1, 3, 6 and 12 months, along with a time-dependent Cox regression. The results revealed that patients experiencing mild to moderate irAEs (grade 1-2) had better ORR, OS and PFS compared with those without irAEs at all landmark. irAEs of grade ≥3, however, demonstrated improved outcomes only in the 12-month landmark analysis.^42^ In a meta-analysis of 51 trials, Hussaini et al.,^32^ found a positive association between the development of irTRAEs and ORR, PFS and OS in non-small-cell lung cancer, melanoma, renal cell carcinoma, urothelial, and head and neck cancers. Notably, irTRAEs grade ≥3 resulted in a better ORR, but not OS.^32^ These results, however, remain open to debate given the heterogeneity of the methods used in each study and the absence of a standardized statistical analysis across all of them. Moreover, the meta-analysis included only one gastrointestinal tumor, and only one study used the combination of anti-PD1 and anti-CTLA4. In view of these results, the association between toxicities and the efficacy of ICIs is still under discussion and requires robust statistical analyses with many patients and events to achieve acceptable statistical power.

On this point, there are also some limitations in our analysis. The absence of a difference in survival could be explained by the lack of power due to a small number of patients with irTRAEs (*n* = 60) or the low number of events. The significant association between ORR and irTRAEs, however, could be biased by the high response rate of MSI tumors to ICI(s). A larger prospective cohort, encompassing different tumor types and combination therapies, is needed to address this question.

Recently, some authors evaluated neoadjuvant ICI in MSI/dMMR CRCs. In the NICHE trial, the combination of nivolumab and ipilimumab in the neoadjuvant setting for MSI/dMMR rectal cancer showed a 100% objective response and 69% of complete histologic response.^43^ Similar results were observed in the NICHE-2 trial for MSI/dMMR colon cancer.^44^ In this way, the Neonipiga trial evaluated neoadjuvant and adjuvant ICIs in MSI/dMMR gastric and GEJ cancers. All patients had R0 surgery and 58.6% had complete histologic response. Moreover, three patients did not have surgery and had a complete endoscopic response.^45^ Elderly patients are particularly vulnerable to post-operative and radiation complications; these results could pave the way for an interesting therapeutic de-escalation.

## Conclusion

Our study represents the first examination of the safety and efficacy of ICIs, whether administered as monotherapy or in combination, in treatment of MSI mGIC in elderly patients. Our analysis unveiled an acceptable toxicity profile across the entire cohort, with no discernible differences between elderly patients and their younger counterparts. Additionally, there was no discrepancy in survival outcomes between the two aged-based groups. By robustly addressing immortal bias through a rigorous statistical analysis, we found no association between survival and the occurrence of irTRAEs. We did, however, observe a noteworthy association between irTRAEs and improved ORR. Validation of our findings requires a larger and prospective cohort.
