# Supplementary material for: Age-related outcomes in MSI/dMMR gastrointestinal cancers treated by immune checkpoint inhibitors and toxicity’s impact on efficacy: an immunoMSI cohort study
Source: ESMO Gastrointest Oncol. 2024 Apr 30;4:100047. doi: 10.1016/j.esmogo.2024.100047 (PMC12836564; doi:10.1016/j.esmogo.2024.100047)
Supplement: Supplementary data [file mmc1.docx]

**Supplemental**

**Supplemental Table 1: Objective response rate according to age**

| Response | All cohort  N = 201 (%) | Patients < 75 years  N = 177 (%) | Patients ≥ 75 years  N = 24 (%) |
| --- | --- | --- | --- |
| Progression | 59 (29,3) | 53 (29,9) | 6 (24) |
| ORR   - Partial Response - Complete Response | 129 (64,1)  72(35,8)  57 (28,3) | 111 (62,7)  59(33,3)  52(29,3) | 18 (75)  13 (54,1)  5 (20,8) |

**p = 0.26 (Fisher exact test);** stable disease: 13 pts in the entire cohort, and 13 in the group < 75 years

**Supplemental Table 2. ORR according to the occurrence of irTRAEs**

| **Response** | **irTRAEs +**  ***(n* = 60)** | **irTRAEs -**  **(*n* = 141)** |
| --- | --- | --- |
| Progression, *n (%)* | 9 (15) | 50 (35) |
| ORR  PR  CR | 46 (77)  27(45)  19 (32) | 83(59)  45(32)  38(27) |

*P* = 0.0012 (Fisher exact test); 5 stable diseases (8%) in irTRAE + and 8 stable diseases (6%) in irTRAE-

To avoid the bias due to type of treatment (monotherapy / combination of ICI) a linear regression was used. The association between ORR and occurrence of irTRAEs was still significant with an Odds Ratio of 2.64 CI 97.5% 1.20-6.22, *P* = 0.021.

ORR, overall response rate, PR, partial response; CR, complete response

**Supplemental Figure 1: Time to the onset of the first irTRAE**


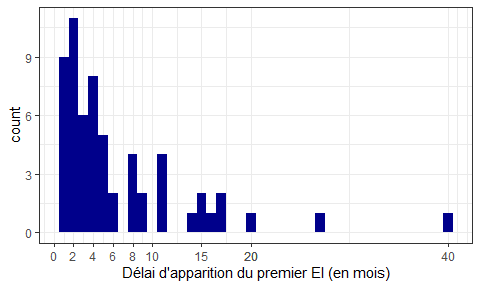


**Supplemental Figure 2 : PFS and OS in the entire cohort**


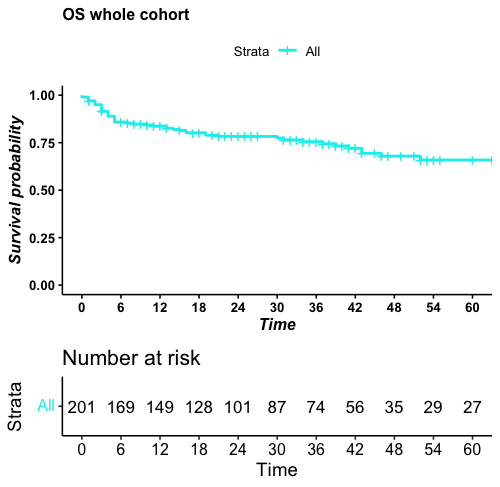

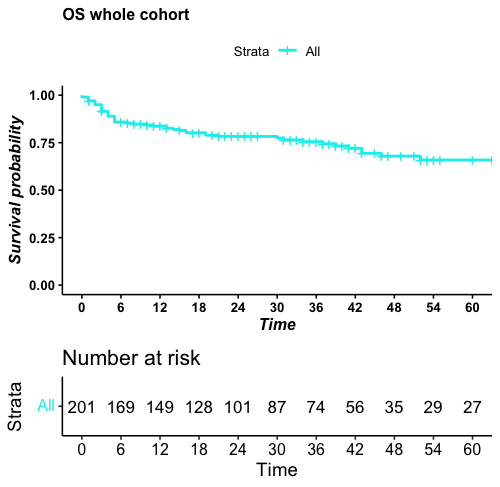


**OS**


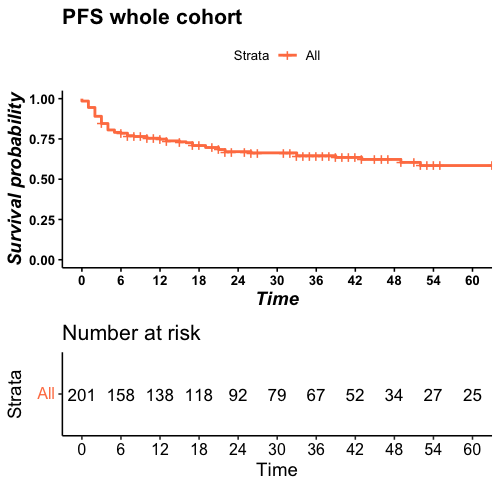

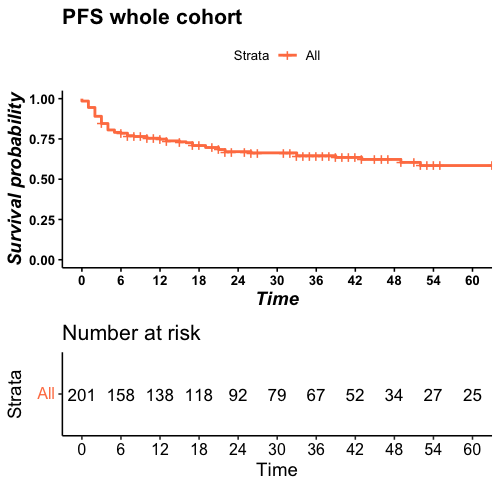


**PFS**
